# CMV70-3P miRNA contributes to the CMV mediated glioma stemness and represents a target for glioma experimental therapy

**DOI:** 10.18632/oncotarget.11175

**Published:** 2016-08-10

**Authors:** Ilya V. Ulasov, Natalya V. Kaverina, Dhimankrishna Ghosh, Marya A. Baryshnikova, Zaira G. Kadagidze, Apollon I. Karseladze, Anatoly Y. Baryshnikov, Charles S. Cobbs

**Affiliations:** ^1^ Swedish Neuroscience Institute, Center for Advanced Brain Tumor Treatment, Seattle, WA, 98122, USA; ^2^ Institute of Experimental Diagnostics and Therapy of Tumors, N.N. Blokhin Russian Cancer Research Center, Moscow, 115478, Russia; ^3^ NN. Blokhin Cancer Research Center, RAMN, Moscow, 115478, Russia; ^4^ Current employment: Division of Nephrology, University of Washington, Seattle, 98109, WA, USA

**Keywords:** brain tumor, cytomegalovirus, glioma stem cells, miRNA

## Abstract

Glioblastoma multiforme (GBM) is a rapidly progressive brain tumor with a median survival of 15–19 months. Therapeutic resistance and recurrence of the disease is attributed to cancer stem cells (CSC). Here, we report that CMV70-3P miRNA encoded by CMV increases GBM CSC stemness. Inhibition of CMV70-3P expression using oligo inhibitors significantly attenuated the ability of primary glioma cells to proliferate and form neurospheres. At the molecular level, we show that CM70-3P increases expression of cellular *SOX*2. Collectively, these findings indicate that CMV70-3P is a potential regulator of CMV- mediated glioma progression and cancer stemness.

## INTRODUCTION

Glioblastoma (GBM) is a WHO grade-IV brain cancer with a median survival of 15–18 months, making it the deadliest of all brain cancers. Although surgical removal of the tumor mass followed by radiation and chemotherapy improves patient survival, tumor recurrence remains a concern [1, 2]. Recent investigations indicate that a sub-population of tumor cells called cancer stem cells (CSCs) is responsible for the recurrence and therapeutic resistance in GBM [3, 4]. These CSCs possess self-renewal and proliferative capabilities similar to healthy stem cells and can increase the development of mutations, deletions and other genetic abnormalities throughout the tumor. There is considerable interest in identifying CSC promoting factors so that effective therapeutic strategies could be designed in the future. While genetic and micro-environmental elements play an important role in the survival and maintenance of CSCs, new evidence suggesting a role of CMV in cancer stemness has begun to emerge [5]. A direct association of CMV with glioma stem cells expressing CD133 marker has recently been evaluated [5, 6]. Our group has shown the presence of CMV proteins in GBM tissues [7]. CMV gene products such as pp71 [8] and IE1 [9] have been known to be responsible for tumor angiogenesis and invasion. Additionally, signaling pathways possibly deregulated by CMV may activate glioma pathogenesis [10, 11]. Importantly, STAT3 and AKT pathways can be stimulated by CMV [12] in GBM cells, and CMV can accelerate gliomagenesis in a mouse model by induction of STAT3 in GBM CSC [13].

Posttranscriptional viral gene expression could be mediated through the expression of short- noncoding RNA-microRNA. Recent bioinformatical analysis of the CMV genome confirms the presence of 20–22 bp mRNA molecules with mRNA target function [14]. Through the formation of protein complex with RISC, viral microRNAs regulate expression of target mRNA and thus translation of specific proteins [15]. The evidence collected so far suggest that most viral microRNAs are highly expressed during productive infection of purified monoyctes [16]. In contrast to these findings, in the brain tumor cells [17, 18], CMV expresses limited quantities of viral mRNA, miRNA and/or DNA expressions [19, 20]. However, during latent infection, cytomegalovirus, however, promotes expression of cellular and viral genes which may help to establish a long-lasting infection via producing viral glycoproteins and microRNAs [16]. Dolken *et al*. experimentally showed that CMV microRNAs are involved in the horizontal transmission and persistence of viral particles [21], thereby confirming, their pro-oncogenic potential of miRNAs coding by genome.

The hypothesis that CMV induced viral microRNA is a part of CMV induced pathogenesis is strongly supported by the observation that latent CMV infection requires the expression of several types microRNAs [22–24]. One attractive viral microRNA that produced during viral latency is CMV70-3P. CMV70-3P is known to be upregulated during infection of THP-1 human monocytes with CMV. Whereas production of several microRNAs during CMV latency has some effect on viral replication, no involvement of CMV70-3P is detected. Although the role of CMV70-3P for glioma progression is under investigation, it is known that CMV70-3P blocks cellular apoptosis via inhibition of BAX modulator gene (MOAP1) [25]. Given the fact that CMV persistence of GSC is associated with high expression of SOX2 [17, 26] and induction of neurospheres [5], in this report we investigated the link between CMV70-3P microRNA and activation of glioma stemness.

## RESULTS

### Evidence of cmv70-3p miRNA present in the GBM tissues

**Figure 1:**
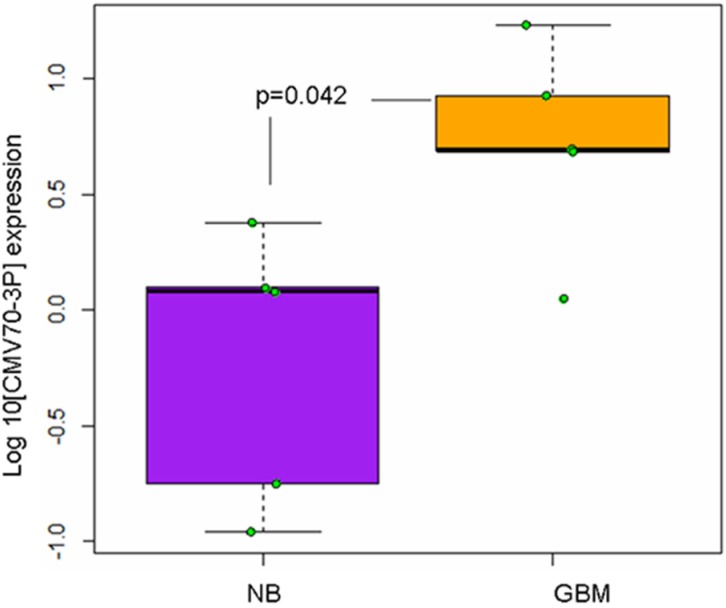
Cytomegalovirus expresses viral miRNAs in the neoplastic tissue of glioma patients Reverse transcriptase qPCR analyses of CMV70-3p in normal brain (NB) and high-grade glioma (GBM) specimens. Reverse transcription with stemloop and 5S RNA primers as the internal control, was carried out as described by Shen et al. [24]. Compared with non-neoplastic brain cells (NB, *N* = 5, magenta box), the level of CMV70-3p microRNA was significantly increased in glioma specimens (GBM, *N* = 5, yellow box). Each real-time PCR assay was performed in triplicate. *P* value = 0.042, one tail *T* test.

Since that various cellular microRNAs play an important role in tumor progression through the regulation of cellular apoptosis [27], invasion [28], autophagy [29] and stemness [30, 31], we hypothesized that CMV70-3P microRNA might also play a role in glioma progression and promote anti-apoptotic function. We analyzed the expression of CMV encoded microRNAs in the human normal brain-like (NB) and GBM cancer specimens (GBM1, GBM2, GBM8, GBM10 and GBM13). Of note, these GBM patient-derived cells were previously characterized for the presence of CMV glycoprotein IE1 [32]. As shown in Figure 1, quantitative PCR analyses of primary specimens reveals that primary glioblastoma cells exhibit high-level expression of CMV70-3P microRNA compared to normal samples (*p* = 0.042, *T* test one tail analyses). Specifically, there was more than 10-fold expression of CMV70-3P microRNA in the GBM tissue relative to normal brain samples, suggesting that CMV70-3P can be a contributing factor for GBM.

### CMV70-3P miRNA expressed in glioma stem cells

**Figure 2:**
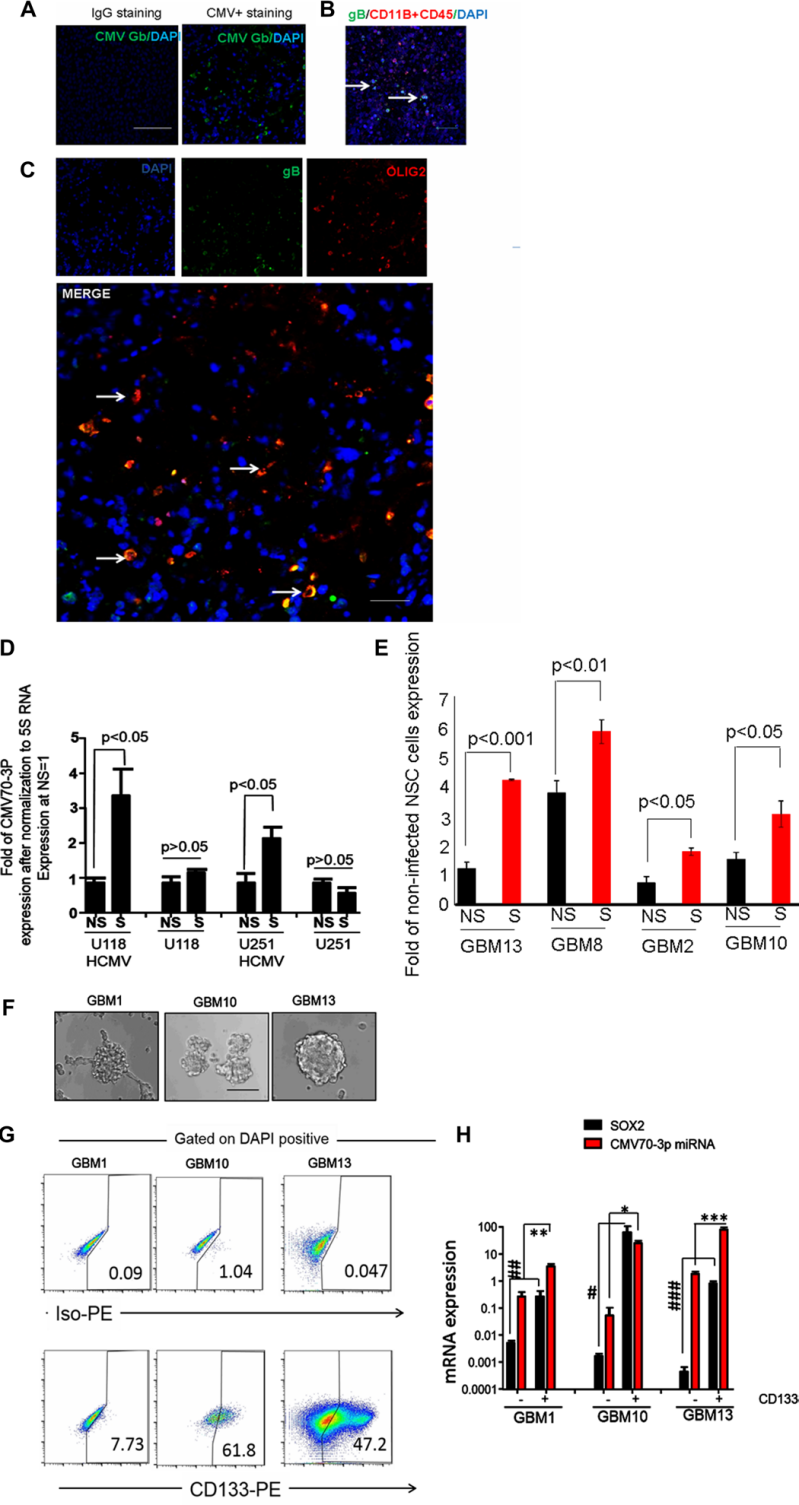
CMV70-3P is enriched in GSC Olig2+ population of GBM contains cytomegalovirus gB. Immunofluorescent staining of primary GBM2 paraffin-embedded sections was conducting using gB (**A**), CD11B, CD45 and gB antibodies (**B**) or gB, Olig2 and DAPI (**C**). Presence of CMV in GBM samples was determined using FITC conjugated gB antibodies. Representative image was presented at part (A). Confocal micrographs of co-localization between DAPI(blue) and gB(green) at part “B” or Olig2 (red) and gB(green) in part “C” was determined. White arrows show areas with green expression (part B) or yellow co-localization (part C). Nuclei were counterstain with DAPI. Magnification ×400, scale 50 μm; (**D**) Expression of CMV70-3P in differential (NS) and glioma stem cells (S) conditions. U251 bearing CMV, U118 infected with CMV cells and primary GBM were growing in the presence of B27, FGF and EGF(“S” condition) or media supplemented with 10%FBS (“NS”-condition) were used for RNA isolation and assessment of CMV70-3P via real-time PCR. Experiment was performed twice in triplicates; (**E**) Relative expression of CMV70-3P was quantified using real-time PCR and later normalized to expression of 5S RNA. Data presented as a fold expression to the level at non-infected human neural cells (NSC, negative control). NS and S –primary cells propagated in non-stem cells or stem cells conditions. GBM2, GBM8, GBM10 and GBM13-pateint-derived glioma cells; Patient GSC are enriched with CMV70-3P. Patient-derived GBM2, GBM10 and GBM13 cells (**F**) were grown in stem cells conditions for 2 weeks before their separation using CD133 marker (**G**). Percentage of CD133 positive cells is presented for each GBM sample; (F) GSC isolated from GBM surgical specimens formed neurospheres as shown in representative images. Magnification ×200, scale 20 μ. Total RNA isolated from CD133 positive and CD133 negative were used for quantitative PCR using primers, which recognize human *SOX*2 and CMV70-3P (**H**). *, **, ***P value expression of CMV70-3P in CD133 positive vs expression in CD133 negative cells. ^#^, ^##^. ^###^*P* value expression of *SOX*2 in CD133 positive vs expression in CD133 negative cells.

To determine the expression of CMV in the various glioma specimens, we stained primary GBM specimens with CD11B (a marker of blood cells and macrophages), CD45 (a marker of hematopoietic cells), antibodies against CMV gB glycoprotein and Olig2 (stem cell markers). We noticed that some gB positive cells (green color, Figure 2A) exhibit CD11B-negative/CD45-negative phenotype (green color, Figure 2B), and the majority of gB positive cells represent a OLIG2-positive subclass of glioma cells (yellow signal of colocalization, Figure 2C and Supplementary Figure S1A) or exhibit strong expression for SOX2 (Supplementary Figure S1B). To further analyze the potential role of CMV70-3P in gliomagenesis, we infected U251 and U118 cells with CMV (5 MOI), and maintained them in monolayer and as spheres to promote self-renewal. As shown in Figure 2D, quantitative assessment of CMV70-3P microRNA revealed the strong presence of CMV70-3P in the stem cell conditions (1.5- and 2.3-fold vs a value of the U118 and U251 cells growing in the DMEM with 10%FBS conditions). Similar results were obtained using patient-derived glioma cells (Figure 2E). To determine if CMV70-3P is associated with known cancer stem cell marker CD133 expression, we isolated primary GBM cells (Figure 2F) as CD133 positive and CD133 negative populations and determined their ability to form neurospheres *in vitro*. We observed higher prevalence of CD133-positive cells in the neurospheres of patient-derived glioma cells (61.8% in GBM10, 7.73% in GBM1 and 47.2 in GBM13 relatively) (Figure 2G). Moreover, CMV70-3P expression was significantly higher in the CD133 positive population (12.65, 4.5 and 51.09 fold relatively) relative to CD133 negative population of GBM10, GBM1 and GBM13 tumor cells (Figure 2H). Of note, we also observed high expression of CMV70-3P, correlated with known stem cell factor *SOX*2.

### CMV70-3P regulates cancer stemness of CD133 positive cells

**Figure 3:**
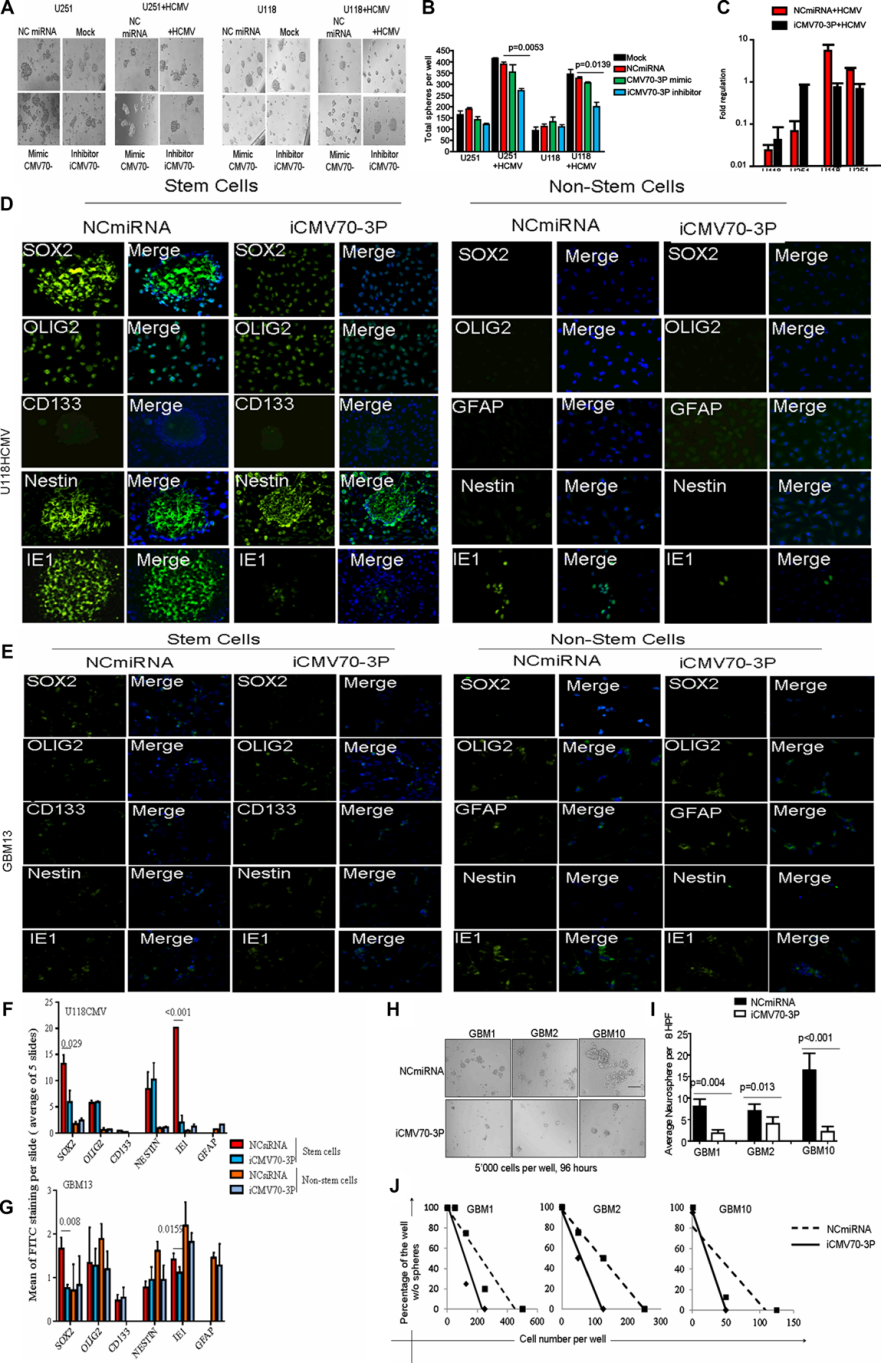
CMV70-3P regulates glioma stemness Growing in stem cell conditions, U251CMV and U118CMV were transfected with NCmiRNA, mimic(mimic CMV70-3P) or inhibitor of CMV70-3P microRNA (each 20 nM) followed by 24 hours incubation in NSA-A NeuroCult^TM^ media supplemented with EGF and bFGF(stem cell condition). Forty-eight hours after transfection, total neurospheres were assessed as described in Materials and Methods. (**A**) Representative photomicrographs (original magnification at ×10) of neurosphere in presence of NCmiRNA, mimic CMV70-3P or inhibitor of CMV70-3P. The total amount of neurospheres (**B**) was plotted and values represents average ± SE. *P* values are indicated in the Figures. There was significant inhibition of neurspheres formation (*p* = 0.0053 and *p* = 0.0139) detected in the U251CMV and U118CMV cells treated with iCMV70-3P (blue bar) vs NCmiRNA (red bar) respectively; (**C**) Real time PCR of SOX2 gene expression detected in the mock or CMV infected U251 and U118 cells after transfection them with NCmiRNA or inhibitor CMV70-3P microRNA (each 20 nM) followed by 24 hours incubation in NSA-A NeuroCult media supplemented with B27, EGF and bFGF. Total RNA was isolated and real-time PCR was performed. ( *p* < 0.05). CMV70-3P expression vs NCmiRNA; Confocal images of U118 glioma cells bearing CMV (**D**) or GBM13 (**E**), and then transfected with oligo inhibitor against CMV70-3P(iCMV70-3P) or control(NCmiRNA) and cultured in the presence of B27/FGF2/EGF(Stem Cells, “S” condition) or 10%FBS (non-stem cells, “NS” condition) and image quantification using Image J software (**F** and **G**). NCmiRNA (non-coding miRNA) or iCMV70-3P transfected U118CMV glioma cells were stained using *NESTIN* (green), *SOX*2 (green), *OLIG2* (green) and *CD133* (green), cytomegalovirus IE1 (green) or GFAP (green) antibodies. Nuclei counterstained with DAPI (blue). CMV70-3P inhibition suppresses expression of *SOX*2 and IE1 in stem cells condition. Magnification ×200, Scale bars represent 20 μ. Five slides per each condition, treatment or cell type were subjected to mean signal quantification. Average of signal was plotted; CMV improves clonogenecity of glioma cells. Stem cells grown and then iCMV70-3P or NCmiRNA transfected GBM1, GBM2 and GBM10 cells were seeded into 96-well plates with defined densities (10–1000 cells per well) and resulting wells with neurospheres (**H**) were scored (**I**) 96 hours after transfection at density of 500 cells per well. Presence of CMV70-3P inhibitor suppress formation of secondary neurospheres at GBM1 (*p* = 0.004), GBM2 (*p* = 0.013) and GBM10 (*p* < 0.001) cells; (**J**) Distribution of the cells transfected with iCMV70-3P determined 14 days after transfected using GBM1, GBM10 and GBM13 patient-derived glioma cells. Limited dilution was calculated as described elsewhere [54].

To determine CMV70-3P's contribution in promoting GBM stemness, we treated CMV infected U251 and U118 glioblastoma cell lines with NCmiRNA (control) or CMV70-3P oligo inhibitor. As seen in Figure 3A, CMV infected U251 and U118 glioblastoma cell lines transfected with CMV70-3P inhibitor exhibited fewer neurospheres (350.2 ± 59.9 (*p* = 0.0053 vs NCmiRNA)) compared to NCmiRNA or mimic (370.2 ± 41.3 neurospheres) (Figure 3B). Similar trends were also observed in U118 cells. Delivery of CMV70-3P inhibitor (iCMV70-3P) significantly suppressed neurosphere formation from 325.5 ± 35.7 (NCmiRNA) and 305.5 ± 15.1 (CMV70-3Pmimic) to 190.5 ± 21.2 (iCMV70-3P, *p* < 0.05) and negatively affected cell viability (Supplementary Figure S2). To determine whether the stem cell marker *SOX*2 is a direct target for CMV70-3P, we investigated whether delivery of CMV70-3P inhibitor could abrogate *SOX*2 expression. Indeed, quantitative PCR analysis indicated that delivery of CMV70-3P microRNA oligo inhibitor to GSC decreased the expression of *SOX*2 following by CMV infection (Figure 3C) and led to ∼20% reduction of human SOX2 promoter activity (Supplementary Figure S3) in the presence of CMV70-3P microRNA expression. To validate whether inhibition of CMV70-3P decreases *SOX*2 protein expression, we used GBM13 patient-derived glioma cells (CMV positive) and U118 cells infected with CMV and then transduced with CMV70-3P or NCmiRNA. The transfected glioma cells grown in differentiated (DMEM with 10% FBS allows to maintain mixture of cells with various lineage, Supplementary Figure S4) and stem cell conditions (NSA media supplemented with N2, B27, EGFFR and FGF) were stained using *CD133*, *NESTIN, GFAP* and *OLIG*2 markers. We found that the delivery of CMV70-3P inhibitor significantly inhibited *SOX2* expression in U118CMV infected and GBM13 glioma stem cells (*p* < 0.029 and 0.008, respectively Figure 3F and 3G) without altering the expressions of *CD133*, *NESTIN* and *OLIG*2. In non-stem cell conditions we did not see significant changes compared to NC control miRNA transfection (Figure 3D). Similar data were obtained using GBM13 cells infected with CMV (Figure 3E). To evaluate the formation of secondary neurospheres during CMV70-3P suppression, cells from patient-derived glioma neurospheres (GBM13) were transfected with NCmiRNA or CMV70-3P inhibitor and were grown in stem cell conditions (Figure 3H). We observed that the delivery of CMV70-3P inhibitor diminished formation of secondary neurospheres in GBM1, GBM2 and GBM10 glioma cells (Figure 3I, all *p* value < 0.05). Moreover, 14 days after transfection we also detected decreased level of neuropsheres using limited dilution assay (Figure 3J), suggesting that glioma long-term culture does support neurosphere formation after CMV70-3P inhibition. These data highlight the role of CMV70-3P microRNA in GBM stemness promoted through CMV infection.

### Inhibition of CMV70-3P abrogates migration and invasion of glioma stem cells

**Figure 4:**
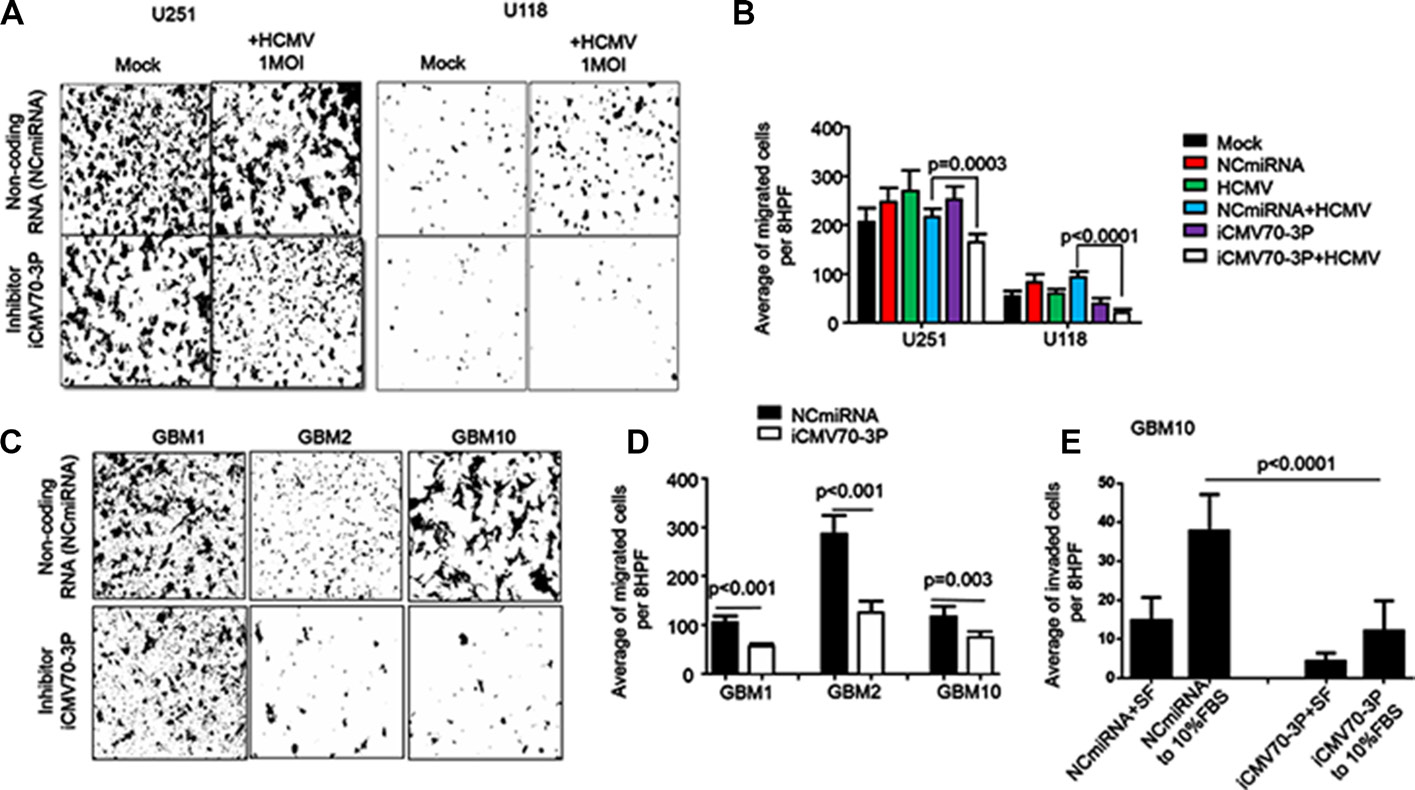
Enforced inhibition of CMV70-3P reduces migration and invasion of glioma cells Mock or CMV infected U251 and U118 human glioma cells, patient derived GBM (GBM1, GBM10 and GBM13) persistent with CMV were transfected with either control (NCmiRNA) or CMV70-3P microRNA inhibitor (iCMV70-3P) and 48 hours later were loaded to the upper chamber of Boyden plate to evaluate cancer cells migration (**A**, **B**, **C** and **D**) or invasion (C). Experiment was performed twice in duplicates and representative photomicrographs (A and C) from one of experiment as well as quantitative analyses (B and D) of migrated or invaded (**E**) cells were plotted. Inhibition of CMV70-3P significantly decrease migration of CMV infected cells (B, blue bars) vs CMV infected cells and then treated with NCmiRNA(white bar, *p* = 0.0003(U251) and *p* < 0.001(U118) cells. Similar data were obtained from patient-derived CMV infected GBM1 (*p* < 0.01), GBM2 (*p* < 0.01) and GBM10 (*p* = 0.003). iCMV70-3P inhibits GBM10 invasion towards 10%FBS chemoattractant (E, *p* < 0.0001). Image magnification ×100. Scale bars, 50 μm. Bars: Mean ± SE.

To determine the role of CMV70-3P expression in invasion, we treated GBM cells (prior infected with CMV) with mock or inhibitor. In the migration test, the mean number of mock or NCmiRNA transfected U118 and U251 pretreated with CMV were 415.5 ± 7.77 or 390 ± 14.14 (U251cells) and 345 ± 36.77 or 326 ± 12.02 (U118 cells) respectively (Figure 4A). After delivery of iCMV70-3P to the CMV infected cells, migration of glioma cells significantly decreased from 415.5 to 272 ± 19.67 (*p* < 0.0003, for U251) cells and 345 to 199 ± 36.46 (*p* < 0.0001) for U118 cells, as shown in Figure 4B. Similar results were obtained using GBM1 (*p* < 0.001), GBM2 (*p* < 0.01) and GBM10 (*p* = 0.003) cells (Figure 4C and 4D). Matrigel invasion assay also demonstrated that delivery of iCMV70-3P suppressed invasion of patient derived glioma cells (Figure 4E). In particular, we detected significant suppression of cell invasion by iCMV70-3P (12.07 ± 7.82) vs GBM10 patient-derived glioma cells transfected with NCmiRNA (36.57 ± 12.72, *p* < 0.0001). Taken together these results indicate that migration and invasion of GBM stem cells can be induced by CMV70-3P miRNA.

## DISCUSSION

Despite the improvement in treatment options, there has been little or no improvement in the survival of GBM patients. Tumor recurrence and therapeutic refractoriness of GBM are known to be linked with the presence of cancer stem cells (CSCs). There is considerable interest in understanding how cancer stem cells can perpetuate the self-renewal and proliferative capabilities of GBM. In the work presented here, we have shown that CMV, through the expression of CMV70-3P, influences glioma stemness. In general, the role of viruses in promoting cancer is well established [33–36]. Since the discovery of CMV in GBM [7], a number of reports describing the potential utility of antiviral therapy and vaccines has been published [37]. This data along with a study conducted by Price *et al*. [13] suggest that further understanding of the molecular biology of CMV as a tool in the investigation of glioma progression may offer advantages for GBM patients.

In the past we have shown that CMV persists inside glioma stem cells and can promote the genesis of GBM by decreasing autophagy in patient-derived glioma stem cells [11, 32]. Indeed, we found that real-time PCR analyses of primary GBM samples revealed a direct association of CMV70-3P with GBM, suggesting a role for CMV in cancer development.

Since GSCs promote the development of GBM, we sought to determine whether CMV70-3P expression is associated with these cells. Our immunofluorescence staining identified that *OLIG2* positive cells are co-localized with CMV glycoprotein B. These data suggest that a precursor of GSC, *OLIG*2 positive NSC [38], can be a source for CMV persistence in the glioma tissue. Given the ability of *OLIG*2 positive cells to form tumors *in vivo*, we hypothesized that CMV70-3P could be a contributor to self-renewal and tumorigenic potential. Thus, we aimed to identify a possible association of CD133 GSC with CMV70-3P expression. Our findings suggest that the stem cell state (neurosphere) of glioma promotes accumulation of CMV70-3P in artificial CMV infected U118 and U251 cells, and patient derived glioma cells. Furthermore, we observed CMV70-3P expression is higher in CD133 positive relative to CD133 negative cells, suggesting strong association of CMV70-3P with GSCs.

The key finding of the current study is that CMV70-3P promotes stemness of GSCs. It was shown that CMV70-3P, which is encoded by the CMV genome, is expressed during effective and persistent infection of various cells by CMV, helping CMV to maintain its persistence [24]. In addition, sustained CMV-mediated signaling is able to maintain GSCs to drive cancer cell stemness [17]. Taken together, we hypothesize that CMV can direct the progression of stem cells through the expression of stemness markers. We show that in CMV infection of U118 and U251 glioma cells, suppression of CMV70-3P resulted in loss of neurospheres formation and reduced proliferation. Additionally, we observed that CMV70-3P microRNA may exert its effect through upregulation of stem cell factor *SOX2*. Such upregulation of *SOX2* by CMV might be mediated through a broader transcriptional regulation that could be investigated in a separate study in the future. Besides transcriptional regulation, CMV70-3P might regulate the mTOR pathway, which is critical for CMV infection since that signaling pathway was implemented in cell cycle division [39], tumor proliferation [40], invasion [41] and migration [42].

In our study we detected that inhibition of CMV70-3P suppressed tumor invasion and migration. Although it is known that changes in SOX2 expression can negatively affect the ability of tumor cells to migrate and invade [45–48], our data show evidence of new relationship between CMV70-3P inhibition and SOX2 expression besides its regulation thorough transcription [49, 50] and p107 [51]. Despite the fact that CMV70-3P can exhibit off-target effects as shown in Figure 3, overall our data demonstrate that suppression of CMV70-3P significantly decreases tumor cell migration and invasion of glioma cells.

## MATERIALS AND METHODS

### Reagents

Against CMV IE1 (MAB8131, Chemicon) and gB (HCMV37, Abcam), human Actin, *SOX*2, *OLIG*2, *NESTIN* (all GeneTex). Cytomegalovirus strain Towne was obtained from Dr. L. Soroceanu (CPMC, California, USA) and propagated with 3T3 cells using standard technique. CMV contained supernatant was collected at designated time points and titrated using plaque forming unit assay human fibroblasts, and stored at minus 80°C. The Pool of pre-miRNA non- silencing random selected sequence of miRNAs with no homology to any known gene sequence (ON-TARGETplus SMARTPool, L-031117-00-0005), sense (mimic) of CMV70-3P miRNA (MIMIAT0003343), as well as, antisense RNA inhibiting activity of human GAPDH (On-TARGETplus, D-001830-10-05), human NES (On-TARGETplus SMARTPool, L-031117-00-0005), cytomegalovirus CMV70-3P (MIMAT0003343) were synthesized by GE Dharmacon (ThermoFisher Scientific). Sense or antisense transfections were carried out using DharmaFECT transfection reagent (ThermoScientific) according to the manufacturer's instructions using mock or CMV infected cells. After transfections, cells were grown in NS-A NeuroCult media supplemented with B27, bFGF and EGF. Accumulation of control genes upon delivery of antisense oligonucleotides was quantified using real-time PCR primers.

### Primary GBM specimens

Primary paraffin embedded specimens, diagnosed as grade IV were provided by Dr. Apollon Karseladse (Department of Pathology, Cancer Research Center). Glioma specimens were obtained from ongoing craniotomies performed at the N.N. Blokhin Cancer Research Center (Moscow, Russia) and Swedish Neuroscience Institute (Seattle, Wa) under written informed consent.

### Ethics statement

Current studies were approved by institutional Review Board of N.N. Blokhin Cancer Research Center and Swedish Medical Center. All patient derived glioma cells were obtained at the Swedish Neuroscience Institute (Swedish Medical Center, Seattle, Wa) under written informed consent and were characterized before [32].

### Primary cells, glioblastoma cell lines and culture conditions

The U251(U251MG and human glioblastoma) and U118(U-118MG and human glioblastoma) were both obtained from ATCC [American Type Culture Collection, Manassas, Va] and previously characterized earlier [32]. Cells were propagated in MEM supplemented with 10% FBS, 1% penicillin-streptomycin (Life Technologies). To preserve their stemness cells were grown in the NS-A neuroCULT^™^media (Stem Cells, Wa, USA) supplemented with B27, bFGF and EGF (25 ng/ml) as previously described [32]. Patient-derived glioma stem cells (GBM1, GBM2, GBM8, GBM10 and GBM13) were growing in stem cells conditions (NS-A media supplemented with B27, bFGF and EGF) up passage 5.

### Establishment of artificial glioma cells lines with CMV persistence

100,000 of U251 or U118 cells were plated in T75 flasks 24 hrs prior to infection with CMV. The Next day, growth factor media was replaced with MEM supplemented with 1% FBS media containing cytomegalovirus particles (5 MOI per cell, Towne strain). Three days later, the virus-containing media was replaced and MEM supplemented with 5% FBS was added to each flask. When cells reached 90% confluence, equal amounts of mock or CMV infected cells were split among three flasks, one of which received NS-A media supplemented with bFGF and EGF, while the other received 10% DMEM, and the final receiving 5% FBS containing media for future cells propagation. Cells growing either in stem cell conditions or in the presence of 10% DMEM were harvested 48 hours later to isolate total RNA, characterize protein expressions or perform migration and neurosphere assays.

### Neurosphere assay

The neurosphere assay was used as indicator of cell clonogenecity. Briefly, 5,000 cells were plated per well into 96-well plates and keep grown in NS-A media supplemented with bFGF and EGF for 7 days.

### Cell toxicity

Mock or CMV infected glioma U251 and U118 cells were then transfected with ncRNA, mimic or inhibitor of CMV70-3P microRNA. After overnight transfection, cells were grown in the media that supports glioma stemness (NS-A, supplemented with FGF and EGF). Seventy two hours later the cells were harvested, trypsinized and the amount of live/dead cells per each cell type of cells was determined using trypan blue exclusion test. At least 5 replicates per sample were measured per each type of cells or treatment. Data was acquired by Nikon microscope and presented as a fold of live cells.

### Cell migration and invasion assays

U118 and U251 glioma cells mock infected or infected with CMV (Towne strain) were later transfected with ncRNA, mimic and inhibitor of CMV70-3P microRNA (all 20 nM). After overnight transfection in RPMI media, cells were washed with PBS and NS-A media supplemented with bFGF and EGF was added to the well. Twenty four hours later, cells were harvested; counted and 50,000 cells were plated a top of Boyden chambers (Filter 8 μ porous). Compete media, MEM supplemented with 10% FBS was added to the lower chambers and served as chemoattractant. Each experiment was performed in three biological replicates.

### Limiting dilution assay

Tumor cells from GBM patient cells and then transfected with iCMV70-3P or NCmiRNA were enzymatically dissociated into single cells, and then plated into 96 well plates with different densities (20–500 cells per well). Cells were incubated at 37°C for 2 weeks [53]. For the second neurospheres, cells growing in neurospheres after transfection with iCMV70-3P or NCmiRNA were dissociated and plated at the density of 500 cells per well to quantify the impact of CMV70-3P inhibition. At the time of quantification, each well was examined for the formation of neurospheres. For a statistical analysis, the numbers of responded events were plotted and stem cell frequency was calculated using the Extreme Limiting Dilution Analysis software.

### Binding of CMV70-3p to human Sox2 promoter

For the transfection experiment HEK293 cells were cotransfected with luciferase expressed LightSwitch Promoter Reporter GoClone or Sox2 GoClone vectors with NCsiRNA (pcmvMIR-NCsiRNA), iCMV70-3P inhibitor or pcmvMIR plasmid expressing CMV70-3P microRNA(pcmvMIR-CMV70-3P). Luciferase activity was measured 48 hours later using a LightSwitch Luciferase assay. Data presented as fold of mock expression based on 4 replicates per sample.

### RNA extraction, cDNA conversion and real time PCR

Total RNA was extracted using a phenol –chloroform approach. The quantity of RNA was assessed using Nanodrop. cDNA synthesis followed by real-time PCR was performed [24]. The amplified product was sequenced. Real-time PCR was conducted using 2× SybrGreen PCR Master Mix (Applied Biosystems) on ABI7900HT (Applied Biosystems). Relative expressions of miRNA or CD133 were normalized to 5S RNA or GAPDH relatively. Expression levels of *SOX*2 were quantified in triplicate relative to beta-actin using the ΔΔCt method. Data were presented as fold expression vs mock infected cells. The primer set for human *SOX*2 was purchased from www.realtimepcr.com. Sequence for *GAPDH* primers are as follows: 5 GGTTTACATGTTCCAATA-3′ and 5′ATGGGATTTCCATTGATGACAAG-3′.

### Western blotting

Twenty micrograms of total protein was obtained from treated/untreated cells and loaded per lane. To measure protein expressions membranes with immobilized proteins were incubated with the appropriate primary antibodies overnight [32]. The following day, proteins were visualized using species-specific anti-IgG secondary antibodies (Li-Cor Biosciences, Lincoln, NE, USA). Images were acquired using Odyssey Imaging System (Model #1866, Li-COR Biosciences, Lincoln, NE).

### Flow cytometry

Human glioma cells were grown in neurospheres conditions [32]. to promote stemness and subjected to staining with CD133 antibody (Mylteny Biotech). After 1 hour, unbound antibody was removed and cells were analyzed by flow cytometry for CD133 PE expression, using FACScalibur (Becton-Dickenson, Bedford, Ca) at the Fred Hutchinson Cancer Center Flow Cytometry Facility. A minimum of 10,000 events were scored for each sample.

### Immunofluorescence staining of glioma cells growing on coverslips

GBM10 cells were grown on glass coverslips at 80% confluence. After fixation and permeabilization with ice cold methanol, cells were incubated overnight at 4°C with mouse or rabbit antibodies against human Nestin, GFAP, B3-Tubulin and OLIG2 antigens. The next day cells were washed and then incubated with Alexa 568 or 598 fluorochrome-conjugated donkey anti-mouse or anti0-rabbit antibodies for 1 Hr at room temperature. Coverslips were mounted and examined in immunofluorescence microscope.

### Confocal microcopy analysis

For two-dimensional evaluation, 4-μm fresh-frozen sections were stained with immunofluorescent antibodies against CD11B, Olig2, Sox2, CD45 and CMV glycoprotein gB of CMV in combination with DAPI (Invitrogen^™^, Life Technologies, Grand Island, NY). As a negative control, all stainings were performed without primary antibody. Fluorescently labeled species-specific secondary anti–immunoglobulin G (IgG) antibody (Invitrogen^™^, Life Technologies, Grand Island, NY) was applied for visualization of primary antibody. Images were captured with Olympus IX2-UCB DSU spinning disc confocal microscope (Olympus, Center Valley, PA) with an Evolve electron-multiplying charge-coupled device camera (Photometrics, Tucson, AZ). Dual-color immunofluorescence was performed at 600× magnification and a high–numerical aperture (NA, 1.2) oil-immersion objective. Images were acquired and stored in original SlideBook format (Intelligent Imaging Innovations Inc, Denver, CO). After image capture, files were saved in TIFF format. Images were quantified using image J software. Prior to the measurement of mean FITC background was automatically subtracted from each slide prior assessment of fluorescence. Data was plotted and statistical analyses were conducted on average of mean fluorescence per 5 slides per staining.

### Statistical analysis

Student's *t* test was used for data analyses and *p* value < 0.05 was considered significant. All values are shown as mean+/− standard error (SE).

## SUPPLEMENTARY MATERIALS FIGURES



## Abbreviations

GBM: glioblastoma
GSC: glioma stem cells
CMV: human cytomegalovirus
siRNA: anti-sense RNA
miRNA: micro RNA

## References

[1] Liu B, Pang B, Liu H, Arakawa Y, Zhang R, Feng B, Zhong P, Murata D, Fan H, Xin T, Zhao G, Liu W, Guo H (2015). High mobility group A1 expression shows negative correlation with recurrence time in patients with glioblastoma multiforme. Pathol Res Pract.

[2] Auffinger B, Spencer D, Pytel P, Ahmed AU, Lesniak MS (2015). The role of glioma stem cells in chemotherapy resistance and glioblastoma multiforme recurrence. Expert Rev Neurother.

[3] Bao S, Wu Q, Sathornsumetee S, Hao Y, Li Z, Hjelmeland AB, Shi Q, McLendon RE, Bigner DD, Rich JN (2006). Stem cell-like glioma cells promote tumor angiogenesis through vascular endothelial growth factor. Cancer Res.

[4] Chua C, Zaiden N, Chong KH, See SJ, Wong MC, Ang BT, Tang C (2008). Characterization of a side population of astrocytoma cells in response to temozolomide. J Neurosurg.

[5] Soroceanu L, Matlaf L, Khan S, Akhavan A, Singer E, Bezrookove V, Decker S, Ghanny S, Hadaczek P, Bengtsson H, Ohlfest J, Luciani-Torres MG, Harkins L (2015). Cytomegalovirus Immediate-Early Proteins Promote Stemness Properties in Glioblastoma. Cancer Res.

[6] Fornara O, Bartek J, Rahbar A, Odeberg J, Khan Z, Peredo I, Hamerlik P, Bartek J, Stragliotto G, Landazuri N, Soderberg-Naucler C (2015). Cytomegalovirus infection induces a stem cell phenotype in human primary glioblastoma cells: prognostic significance and biological impact. Cell Death Differ.

[7] Cobbs CS, Harkins L, Samanta M, Gillespie GY, Bharara S, King PH, Nabors LB, Cobbs CG, Britt WJ (2002). Human cytomegalovirus infection and expression in human malignant glioma. Cancer Res.

[8] Matlaf LA, Harkins LE, Bezrookove V, Cobbs CS, Soroceanu L (2013). Cytomegalovirus pp71 protein is expressed in human glioblastoma and promotes pro-angiogenic signaling by activation of stem cell factor. PLoS One.

[9] Cobbs CS (2011). Evolving evidence implicates cytomegalovirus as a promoter of malignant glioma pathogenesis. Herpesviridae.

[10] Cobbs CS, Soroceanu L, Denham S, Zhang W, Britt WJ, Pieper R, Kraus MH (2007). Human cytomegalovirus induces cellular tyrosine kinase signaling and promotes glioma cell invasiveness. J Neurooncol.

[11] Cobbs CS, Soroceanu L, Denham S, Zhang W, Kraus MH (2008). Modulation of oncogenic phenotype in human glioma cells by cytomegalovirus IE1-mediated mitogenicity. Cancer Res.

[12] Cobbs C, Khan S, Matlaf L, McAllister S, Zider A, Yount G, Rahlin K, Harkins L, Bezrookove V, Singer E, Soroceanu L (2014). HCMV glycoprotein B is expressed in primary glioblastomas and enhances growth and invasiveness via PDGFR-alpha activation. Oncotarget.

[13] Price RL, Song J, Bingmer K, Kim TH, Yi JY, Nowicki MO, Mo X, Hollon T, Murnan E, Alvarez-Breckenridge C, Fernandez S, Kaur B, Rivera A (2013). Cytomegalovirus contributes to glioblastoma in the context of tumor suppressor mutations. Cancer Res.

[14] Chen K, Rajewsky N (2007). The evolution of gene regulation by transcription factors and microRNAs. Nat Rev Genet.

[15] Ng KR, Li JY, Gleadle JM (2015). Human cytomegalovirus encoded microRNAs: hitting targets. Expert Rev Anti Infect Ther.

[16] Meshesha MK, Bentwich Z, Solomon SA, Avni YS (2016). In vivo expression of human cytomegalovirus (HCMV) microRNAs during latency. Gene.

[17] Fiallos E, Judkins J, Matlaf L, Prichard M, Dittmer D, Cobbs C, Soroceanu L (2014). Human cytomegalovirus gene expression in long-term infected glioma stem cells. PloS one.

[18] Cobbs CS, Matlaf L, Harkins LE (2014). Methods for the detection of cytomegalovirus in glioblastoma cells and tissues. Methods Mol Biol.

[19] Yamashita Y, Ito Y, Isomura H, Takemura N, Okamoto A, Motomura K, Tsujiuchi T, Natsume A, Wakabayashi T, Toyokuni S, Tsurumi T (2014). Lack of presence of the human cytomegalovirus in human glioblastoma. Mod Pathol.

[20] Baryawno N, Rahbar A, Wolmer-Solberg N, Taher C, Odeberg J, Darabi A, Khan Z, Sveinbjornsson B, FuskevAg OM, Segerstrom L, Nordenskjold M, Siesjo P, Kogner P (2011). Detection of human cytomegalovirus in medulloblastomas reveals a potential therapeutic target. J Clin Invest.

[21] Dolken L, Krmpotic A, Kothe S, Tuddenham L, Tanguy M, Marcinowski L, Ruzsics Z, Elefant N, Altuvia Y, Margalit H, Koszinowski UH, Jonjic S, Pfeffer S (2010). Cytomegalovirus microRNAs facilitate persistent virus infection in salivary glands. PLoS Pathog.

[22] Grey F, Antoniewicz A, Allen E, Saugstad J, McShea A, Carrington JC, Nelson J (2005). Identification and characterization of human cytomegalovirus-encoded microRNAs. J Virol.

[23] Grey F, Meyers H, White EA, Spector DH, Nelson J (2007). A human cytomegalovirus-encoded microRNA regulates expression of multiple viral genes involved in replication. PLoS Pathog.

[24] Shen ZZ, Pan X, Miao LF, Ye HQ, Chavanas S, Davrinche C, McVoy M, Luo MH (2014). Comprehensive analysis of human cytomegalovirus microRNA expression during lytic and quiescent infection. PLoS One.

[25] Babu SG, Pandeya A, Verma N, Shukla N, Kumar RV, Saxena S (2014). Role of HCMV miR-UL70-3p and miR-UL148D in overcoming the cellular apoptosis. Mol Cell Biochem.

[26] Fornara O, Bartek J, Rahbar A, Odeberg J, Khan Z, Peredo I, Hamerlik P, Bartek J, Stragliotto G, Landazuri N, Soderberg-Naucler C (2016). Cytomegalovirus infection induces a stem cell phenotype in human primary glioblastoma cells: prognostic significance and biological impact. Cell Death Differ.

[27] Park EY, Chang E, Lee EJ, Lee HW, Kang HG, Chun KH, Woo YM, Kong HK, Ko JY, Suzuki H, Song E, Park JH (2014). Targeting of miR34a-NOTCH1 axis reduced breast cancer stemness and chemoresistance. Cancer Res.

[28] Paw I, Carpenter RC, Watabe K, Debinski W, Lo HW (2015). Mechanisms regulating glioma invasion. Cancer Lett.

[29] Gwak HS, Kim TH, Jo GH, Kim YJ, Kwak HJ, Kim JH, Yin J, Yoo H, Lee SH, Park JB (2012). Silencing of microRNA-21 confers radio-sensitivity through inhibition of the PI3K/AKT pathway and enhancing autophagy in malignant glioma cell lines. PLoS One.

[30] Bier A, Giladi N, Kronfeld N, Lee HK, Cazacu S, Finniss S, Xiang C, Poisson L, deCarvalho AC, Slavin S, Jacoby E, Yalon M, Toren A (2013). MicroRNA-137 is downregulated in glioblastoma and inhibits the stemness of glioma stem cells by targeting RTVP-1. Oncotarget.

[31] Zhang Y, Kim J, Mueller AC, Dey B, Yang Y, Lee DH, Hachmann J, Finderle S, Park DM, Christensen J, Schiff D, Purow B, Dutta A (2014). Multiple receptor tyrosine kinases converge on microRNA-134 to control KRAS, STAT5B, and glioblastoma. Cell Death Differ.

[32] Ulasov IV, Shah N, Kaverina NV, Lee H, Lin B, Lieber A, Kadagidze ZG, Yoon JG, Schroeder B, Hothi P, Ghosh D, Baryshnikov AY, Cobbs CS (2015). Tamoxifen improves cytopathic effect of oncolytic adenovirus in primary glioblastoma cells mediated through autophagy. Oncotarget.

[33] Huang SH, Xu W, Waldron J, Siu L, Shen X, Tong L, Ringash J, Bayley A, Kim J, Hope A, Cho J, Giuliani M, Hansen A (2015). Refining American Joint Committee on Cancer/Union for International Cancer Control TNM stage and prognostic groups for human papillomavirus-related oropharyngeal carcinomas. J Clin Oncol.

[34] Liu G, Yu FX, Kim YC, Meng Z, Naipauer J, Looney DJ, Liu X, Gutkind JS, Mesri EA, Guan KL (2014). Kaposi sarcoma-associated herpesvirus promotes tumorigenesis by modulating the Hippo pathway. Oncogene.

[35] Bazarbachi A, Cwynarski K, Boumendil A, Finel H, Fields P, Raj K, Nagler A, Mohty M, Sureda A, Dreger P, Hermine O (2014). Outcome of patients with HTLV-1-associated adult T-cell leukemia/lymphoma after SCT: a retrospective study by the EBMT LWP. Bone Marrow Transplant.

[36] Siddiquey MN, Nakagawa H, Iwata S, Kanazawa T, Suzuki M, Imadome K, Fujiwara S, Goshima F, Murata T, Kimura H (2014). Anti-tumor effects of suberoylanilide hydroxamic acid on Epstein-Barr virus-associated T cell and natural killer cell lymphoma. Cancer Sci.

[37] Nair SK, De Leon G, Boczkowski D, Schmittling R, Xie W, Staats J, Liu R, Johnson LA, Weinhold K, Archer GE, Sampson JH, Mitchell DA (2014). Recognition and killing of autologous, primary glioblastoma tumor cells by human cytomegalovirus pp65-specific cytotoxic T cells. Clin Cancer Res.

[38] Chirasani SR, Sternjak A, Wend P, Momma S, Campos B, Herrmann IM, Graf D, Mitsiadis T, Herold-Mende C, Besser D, Synowitz M, Kettenmann H, Glass R (2010). Bone morphogenetic protein-7 release from endogenous neural precursor cells suppresses the tumourigenicity of stem-like glioblastoma cells. Brain.

[39] Li Y, Zhang P, Qiu F, Chen L, Miao C, Li J, Xiao W, Ma E (2012). Inactivation of PI3K/Akt signaling mediates proliferation inhibition and G2/M phase arrest induced by andrographolide in human glioblastoma cells. Life Sci.

[40] Volkers M, Sussman M (2013). mTOR/PRAS40 interaction: hypertrophy or proliferation. Cell Cycle.

[41] Chandrika G, Natesh K, Ranade D, Chugh A, Shastry P (2016). Suppression of the invasive potential of Glioblastoma cells by mTOR inhibitors involves modulation of NFkappaB and PKC-alpha signaling. Sci Rep.

[42] Holand K, Boller D, Hagel C, Dolski S, Treszl A, Pardo OE, Cwiek P, Salm F, Leni Z, Shepherd PR, Styp-Rekowska B, Djonov V, von Bueren AO (2014). Targeting class IA PI3K isoforms selectively impairs cell growth, survival, and migration in glioblastoma. PLoS One.

[43] Molina JR, Hayashi Y, Stephens C, Georgescu MM (2010). Invasive glioblastoma cells acquire stemness and increased Akt activation. Neoplasia.

[44] Wakimoto H, Kesari S, Farrell CJ, Curry WT, Zaupa C, Aghi M, Kuroda T, Stemmer-Rachamimov A, Shah K, Liu TC, Jeyaretna DS, Debasitis J (2009). Human glioblastoma-derived cancer stem cells: establishment of invasive glioma models and treatment with oncolytic herpes simplex virus vectors. Cancer Res.

[45] Wang X, Ji X, Chen J, Yan D, Zhang Z, Wang Q, Xi X, Feng Y (2014). SOX2 enhances the migration and invasion of ovarian cancer cells via Src kinase. PLoS One.

[46] Yang N, Hui L, Wang Y, Yang H, Jiang X (2014). SOX2 promotes the migration and invasion of laryngeal cancer cells by induction of MMP-2 via the PI3K/Akt/mTOR pathway. Oncol Rep.

[47] Yang N, Wang Y, Hui L, Li X, Jiang X (2015). Silencing SOX2 Expression by RNA Interference Inhibits Proliferation, Invasion and Metastasis, and Induces Apoptosis through MAP4K4/JNK Signaling Pathway in Human Laryngeal Cancer TU212 Cells. J Histochem Cytochem.

[48] Wu F, Ye X, Wang P, Jung K, Wu C, Douglas D, Kneteman N, Bigras G, Ma Y, Lai R (2013). Sox2 suppresses the invasiveness of breast cancer cells via a mechanism that is dependent on Twist1 and the status of Sox2 transcription activity. BMC Cancer.

[49] Kallas A, Pook M, Trei A, Maimets T (2014). SOX2 Is Regulated Differently from NANOG and OCT4 in Human Embryonic Stem Cells during Early Differentiation Initiated with Sodium Butyrate. Stem Cells Int.

[50] Vilas JM, Ferreiros A, Carneiro C, Morey L, Da Silva-Alvarez S, Fernandes T, Abad M, Di Croce L, Garcia-Caballero T, Serrano M, Rivas C, Vidal A, Collado M (2015). Transcriptional regulation of Sox2 by the retinoblastoma family of pocket proteins. Oncotarget.

[51] Wu F, Li JQ, Miki H, Nishioka M, Fujita J, Ohmori M, Imaida K, Kuriyama S (2002). p107 Expression in colorectal tumours rises during carcinogenesis and falls during invasion. Eur J Cancer.

[52] Gao H, Teng C, Huang W, Peng J, Wang C (2015). SOX2 Promotes the Epithelial to Mesenchymal Transition of Esophageal Squamous Cells by Modulating Slug Expression through the Activation of STAT3/HIF-alpha Signaling. Int J Mol Sci.

[53] Lee Y, Kim KH, Kim DG, Cho HJ, Kim Y, Rheey J, Shin K, Seo YJ, Choi YS, Lee JI, Lee J, Joo KM, Nam DH (2015). FoxM1 Promotes Stemness and Radio-Resistance of Glioblastoma by Regulating the Master Stem Cell Regulator Sox2. PLoS One.

[54] Joo KM, Jin J, Kim E, Ho Kim K, Kim Y, Gu Kang B, Kang YJ, Lathia JD, Cheong KH, Song PH, Kim H, Seol HJ, Kong DS (2012). MET signaling regulates glioblastoma stem cells. Cancer Res.

