# Supplementary material for: CMV70-3P miRNA contributes to the CMV mediated glioma stemness and represents a target for glioma experimental therapy
Source: Oncotarget. 2016 Aug 10;8(16):25989–99. doi: 10.18632/oncotarget.11175 (PMC5432232; doi:10.18632/oncotarget.11175)
Supplement: Supplementary file 1 [file oncotarget-08-25989-s001.pdf]

CMV70-3P miRNA contributes to the CMV mediated glioma stemness and represents a target for glioma experimental therapy

Supplementary Materials

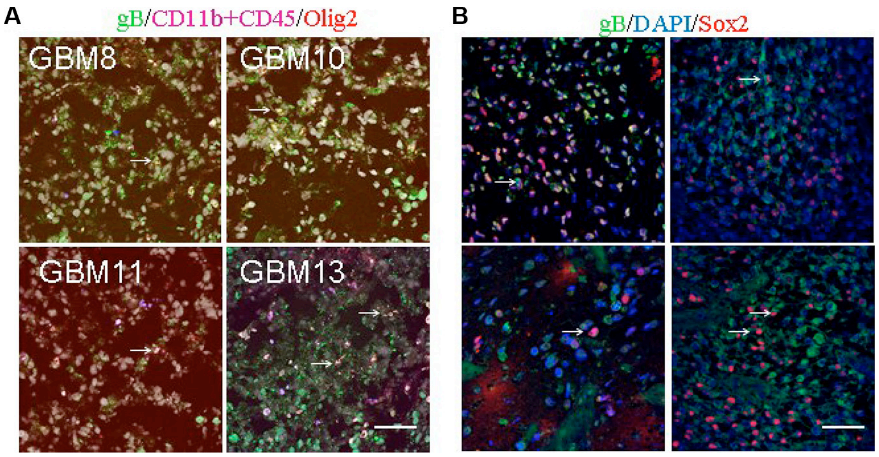

Supplementary Figure S1: Multi-color immunostaining reveals co-localization of CD11B + CD45, CMV and Olig2 (A) or Sox2 (B) expression in the primary glioma cells. A representative (merged) images (600×, scale bar-10 µM) showing co-localization of CMV gB with Olig2 (yellow signal, point arrow), or Sox2 (Gb-cytoplasmic and SOX2-nuclear staining) suggesting that CMV gB is expressed in primary glioma cells of GBM8, GBM10, GBM11 and GBM13; CMV gB pseudo-colored with green; CD11B and CD45 with magenta; OLIG2-with red; Sox2-with magenta and DAPI-with gray (part A) or blue (part B).

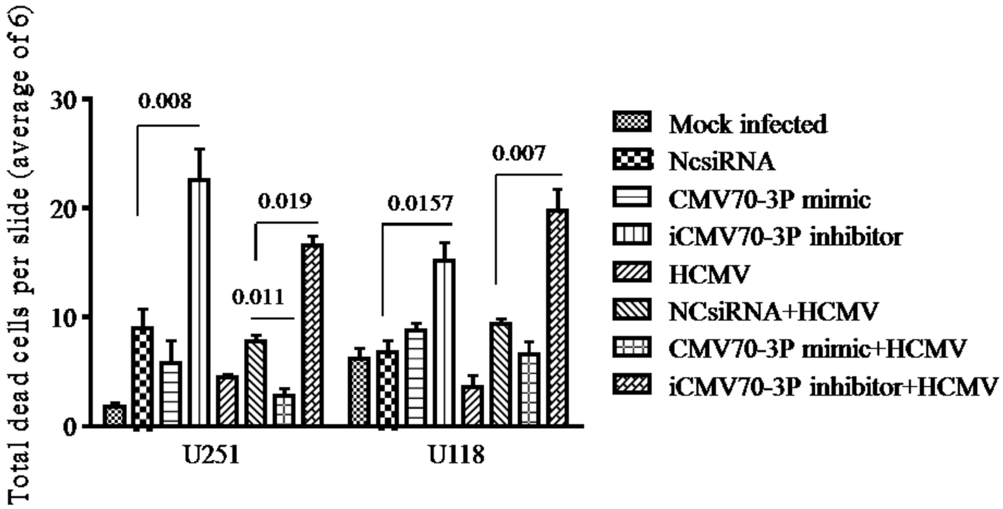

Supplementary Figure S2: Role of CMV70-3P in the cell death. U251 or U118 cells in the presence of HCMV or mock infected were treated with NCsiRNA, CMV70-3 Pmimic or oligo inhibitor CMV70-3P, and 72 hrs later the amount of dead cells per treatment was determined using trypan blue exclusion assay test.

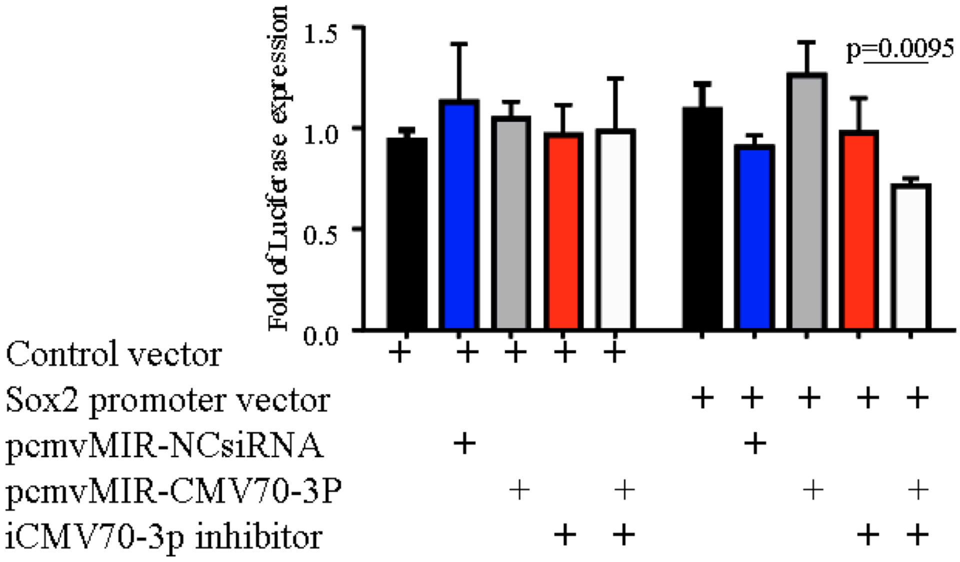

Supplementary Figure S3: CMV70-3P promotes SOX2 expression. The luciferase reporter constructs containing the entire 1.1-kb human SOX2 or 0.6-kb CMV promoters were co-transfected into HEK293 cells with NCsiRNA (pcmvMIR-NCsiRNA), CMV70-3p oligo inhibitor or pcmvMIR expresses CMV70-3P(pcmvMIR-CMV70-3P). Luciferase luminescence readings were obtained 48 hr post-transfection. Relative luminescence was obtained by normalizing the value against respective negative control plasmids. Statistical analyses were done using *T* tests. Error bars represent the mean ± S.D. ($n = 4$).

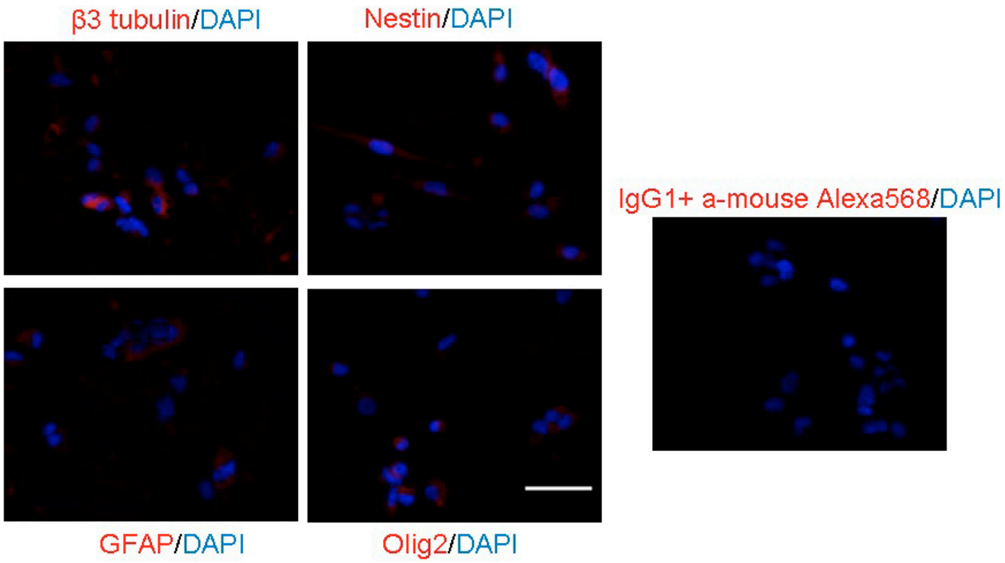

Supplementary Figure S4: Fetal Bovine serum induces differentiation of glioma stem cells. GBM10 patient-derived glioma cells were grown in the lamin-charges glass coverslisps in the presence of DMEM media supplemented with 10% FBS for 10 days. Cells were stained with antibodies against human β3 tubulin, GFAP, Nestin or Olig2 followed by incubation with ALEXA568 conjugated secondary antibodies. Images were collected by immunofluorescence microscope and analyzed using image J software. The magenta signal represents the staining of correspondent protein and blue signal represents the nuclei staining by DAPI. Original magnification 200×.
